# A Novel Approach to Quantify Environmental Risk Factors of Myopia: Combination of Wearable Devices and Big Data Science

**DOI:** 10.1167/tvst.9.13.17

**Published:** 2020-12-10

**Authors:** Lei Li, Longbo Wen, Weizhong Lan, Haogang Zhu, Zhikuan Yang

**Affiliations:** 1 State Key Laboratory of Software Development Environment, Beihang University, Beijing, China; 2 Aier School of Ophthalmology, Central South University, Hunan, China; 3 Aier School of Optometry and Vision Science, Hubei University of Science and Technology, Xianning, China; 4 Guangzhou Aier Eye Hospital, Jinan University, Guangzhou, China; 5 NIHR Biomedical Research Centre for Ophthalmology, Moorfields Eye Hospital NHS Foundation Trust and UCL Institute of Ophthalmology, London, UK

**Keywords:** myopia, working distance, light intensity, spatial data mining, wearable device

## Abstract

**Purpose:**

To develop a practical approach to quantify the exposure to environmental risk factors of myopia.

**Methods:**

In total, 179 children (age, mean ± standard deviation [SD] 9.17 ± 0.52 years) were requested to wear Clouclip, designed to measure working distance (WD) and light intensity (LI), for a whole week. The spherical equivalent refraction (SER) was determined by cycloplegic autorefraction. The raw data of WD and LI were preprocessed through several steps, including data denoising, constructing a two-dimensional WD-LI space, and data sparseness disposing. Weighted linear regression was used to explore the relationship between WD/LI and SER. A novel parameter visual behaviour index (VBI) was developed to summarize the overall impact of WD/LI on SER.

**Results:**

The mean ± SD SER of 179 participants was 0.22 ± 1.18 D. WD and LI were positively associated with SER. However, their magnitude of effect on SER varied with the relative level between them. When WD and LI were split up, the detrimental threshold was approximately 40 cm for WD and 6300 lux for LI. VBI was significantly positively associated with SER (β = 0.0623, *R*^2^ = 0.031, *P* < 0.05).

**Conclusions:**

The current study provides a novel approach to quantify environmental risk factors of myopia. Despite the complexity of the interaction between these risk factors and their impact on SER, this information can be summarized as one single-parameter VBI, which provides a useful tool to investigate the effect of environmental factors on myopia development and progression.

**Translational Relevance:**

We developed a novel approach to quantify environmental risk factors of myopia.

## Introduction

Myopia has emerged as a major health issue in East Asia, because of its increasingly high prevalence in the past few decades (now 80%–90% in young adults)^1^ and the sight-threatening pathologies associated with high myopia.^2^ Although there are dozens of single-nucleotide polymorphisms associated with school myopia,^3^ given the rapid increase of the prevalence in the past 10 years, it seems that the occurrence of myopia is mostly related to environmental factors, such as high educational pressures and less time on outdoor activities.^4^

Nevertheless, to understand the exact role of environmental factors in myopia development, one encounters several challenges. First, one needs to define what specific environmental factors might be responsible for myopia development. Regarding this, recent studies have indicated that the myopiagenic impact of high educational pressures and less time outdoors might be, at least in part, mediated by near-work and light intensity.^5^ Second, one needs to measure these factors in an objective and quantitative manner. In the past, the quantification of these environmental factors mostly has relied on questionnaires,^6^ which have been accused of having recall bias and inaccuracy.^7^ In light of this, several wearable devices, such as HOBO,^8^ Actiwatch,^9^ and Fitsight,^10^ have been adopted to measure the ambient light intensity. Recently, we have developed Clouclip (Glasson Technology Co. Ltd., Hangzhou, China), another wearable device that is able to measure simultaneously the working distance (WD) and eye-level light intensity (LI) with high precision and accuracy.^11^^–^^14^

Although one can analyze the role of these environmental factors in myopia development by relating the WD and the LI data separately to an individual's refractive error, it seems more reasonable to map them into a two-dimensional space with the time series as the coordinate because these two factors always occur simultaneously, and their effect on refractive error may not be simply additive. In this context, the approach of spatial data mining prevails over the conventional approaches to manage numerical and categorical data in that spatial data mining is capable of discovering interesting and previously unknown but potentially useful patterns from large spatial data sets. In addition, the complexities of spatial data and intrinsic spatial relationships in this case limit the usefulness of conventional data-mining techniques for extracting spatial patterns, which include (1) the spatial relationships among variables, (2) the spatial structure of errors, (3) mixed distributions (as opposed to commonly assumed normal distributions), (4) observations that are not independent, (5) spatial autocorrelations among features, and (6) nonlinear interactions in the feature space.^15^

In this report, we have developed a systematic spatial data-mining algorithm that is specialized to analyze the environmental data collected from Clouclip and established a single parameter, the visual behavior index (VBI), which is capable of summarizing the overall effect of the environmental data of a subject. With this, we aimed to provide a practical and easy-to-use approach to quantify the exposure to environmental risk factors of myopia.

## Method

### Participants

The participants for this study were fourth-grade students recruited from three schools. All participants underwent a comprehensive ocular examination, including an ocular health assessment and cycloplegic autorefraction in both eyes. Cycloplegia was induced with three cycles of cyclopentolate 1% (one drop) instilled 5 minutes apart. The cycloplegic status was evaluated by testing the light reflex and pupil dilation 30 minutes after the last administration of cyclopentolate. An autorefractor (model AR-1; Nidek, Aichi, Japan) was used to perform the autorefraction. Spherical equivalent refraction (SER) was calculated as sphere power + 1/2 cylinder power, and the average SER from two eyes was used for the statistical analysis. Myopia was defined as a SER ≤–0.50 D. Only participants with normal ocular health (except the refractive error) and anisometropia of <1.00 D were included in the study.

After the study was explained and before the examination, written consent was obtained from the students and their parents. The study complied with the tenets of the Declaration of Helsinki and was approved by the Aier Eye Hospital Group Ethics Committee (No. IRB2016004).

### Quantification of WD and LI by Clouclip

Clouclip is a 4.7-g weighted device that is attached to spectacle arms to measure WD and LI. The technical specifications and working rationale of Clouclip device have been described elsewhere.^12^ In brief, Clouclip has a built-in infrared distance sensor (measurement range, 15−60 cm) and a light intensity sensor (measurement range, 1−65,536 lux) for detecting the WD and LI in real time. Clouclip is programmed to measure the WD every 5 seconds and LI every 120 seconds. For the individuals who did not wear spectacles, frames without lenses were provided so that Clouclip could be fitted. The participants were required to wear Clouclip throughout the day, except during bathing and sleeping, continuously for 1 week (including 5 weekdays and 2 weekend days) and were encouraged to perform their daily activities as usual during the week. To improve compliance, teachers and parents were asked to check whether the participants were wearing the devices every day at school or at home.

### Data Preprocessing

#### Step 1: Denoising of Raw Data

**Figure 1.**
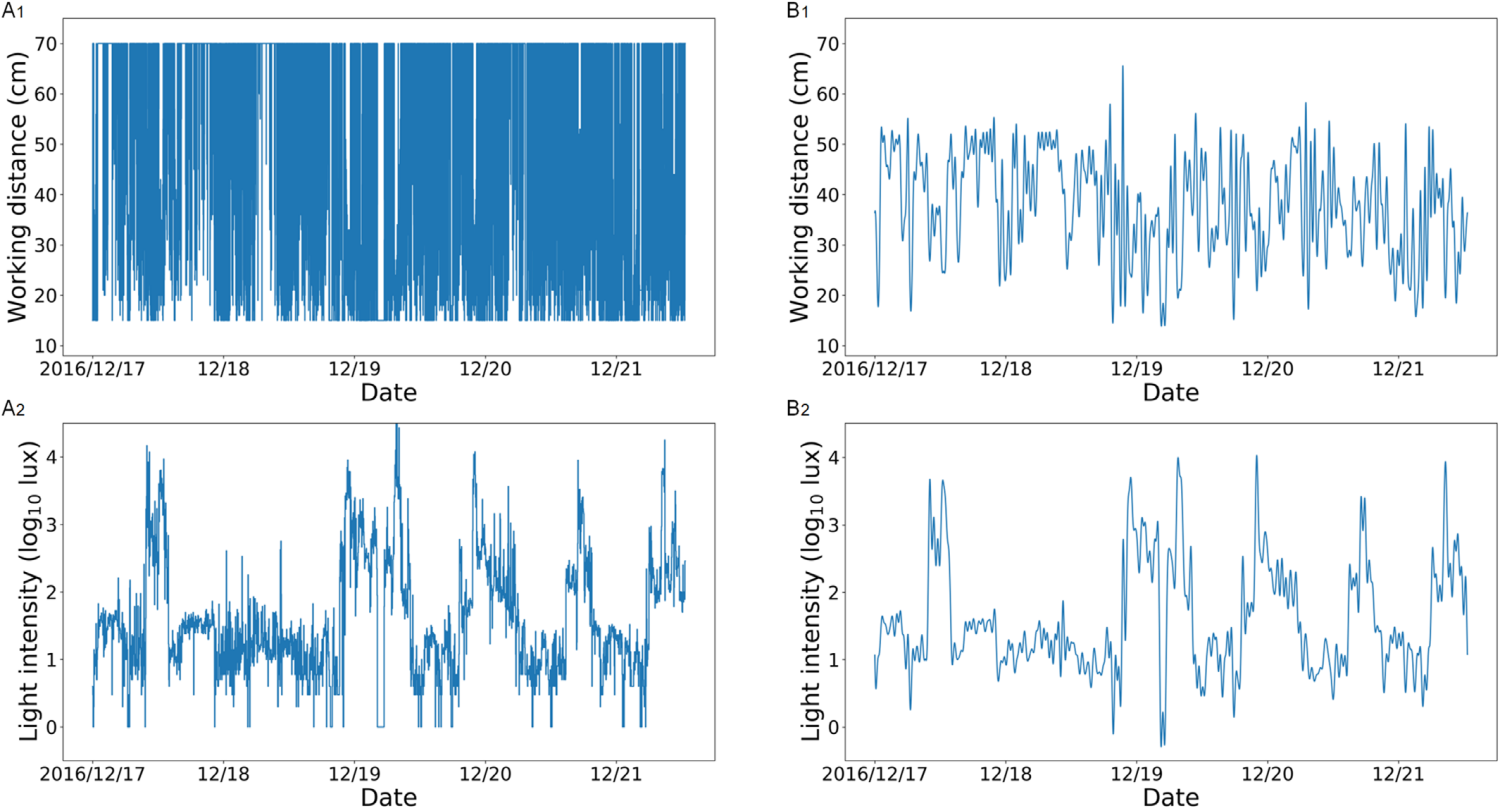
Illustrations of the original WD and LI of one random subject and their FFT- and IFFT-transformed WD and LI. The A_1_ was the original WD series of the subject, and the B_1_ was the FFT- and IFFT-transformed WD series. Similarly, the A_2_ was the original log_10_ of the LI series of the subject, and the B_2_ was his FFT- and IFFT-transformed log_10_ of the LI series.

All raw data, including WD, LI, and the corresponding data collection time points, were downloaded from the cloud platform. As the time series of WD and LI were contaminated with high-frequency variabilities (Figs. 1, A_1_, A_2_), data denoising was performed to filter out noise. Due to the great magnitude of LI, we first used log_10_ to scale the original LI. Then, the fast Fourier transform (FFT) was used as a low-pass filter for both the WD and LI series. As illustrated in Figure 1, for one subject, FFT and inverse FFT (IFFT) were applied to the WD and LI with frequency thresholds to filter out high-frequency variabilities, respectively. Specifically, the WD and LI were transferred from the time domain to the frequency domain by FFT. Then IFFT was used to transfer the segmented frequency spectrums of WD and LI from the frequency domain to the time domain. The filtered WD and LI were smoother than the original WD and LI, and the distributions of the filtered WD and LI were also more explicit (Figs. 1, B_1_, B_2_).

#### Step 2: Creating a Two-Dimensional Space for WD and LI

**Figure 2.**
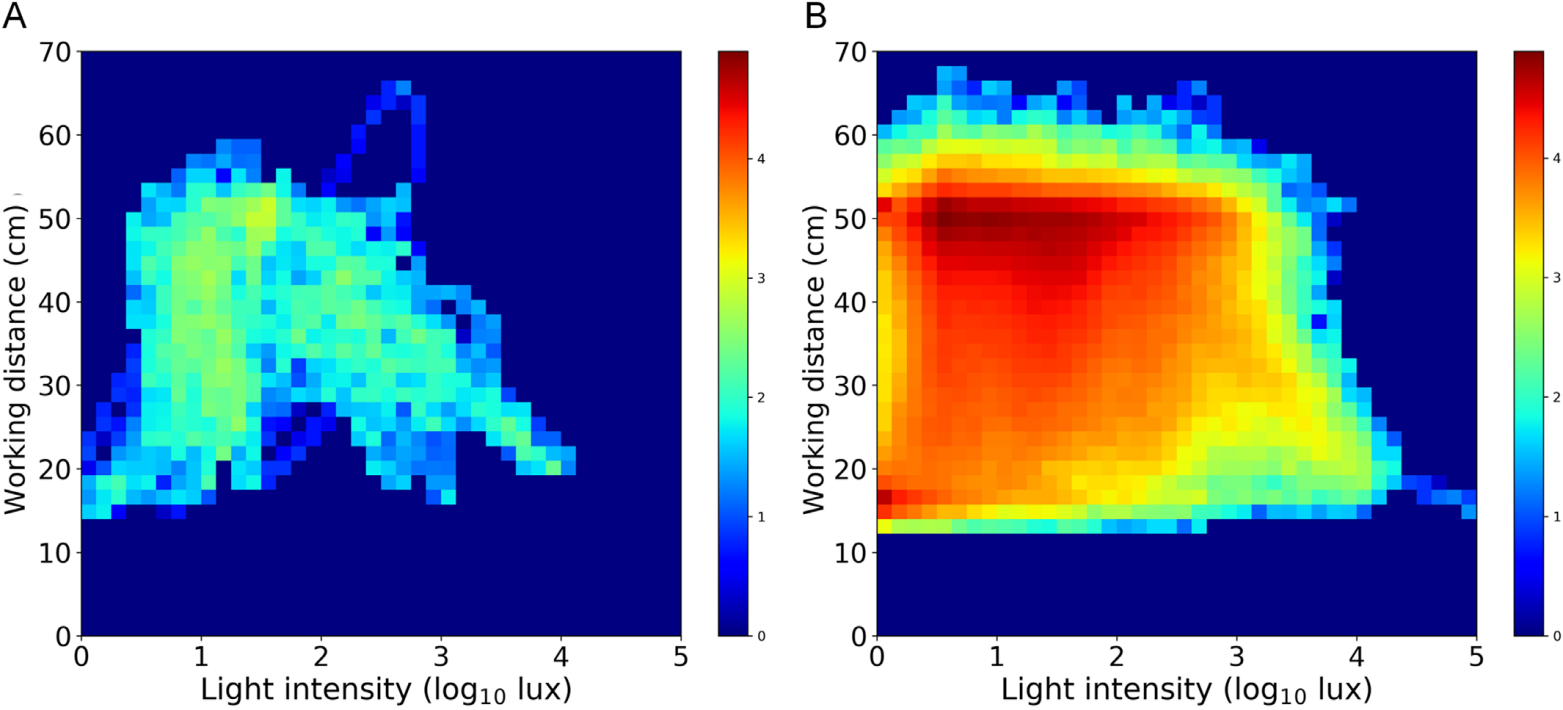
The two-dimensional histogram for one subject (**A**) and all subjects (**B**). For each pixel, the *hotter color* indicates more PoT spent in it, while the *cooler color* indicates less PoT spent in it. The *blue pixels* indicate that there was no PoT in corresponding circumstances.

A two-dimensional space (WD-LI space) was used to present the filtered WD and LI data, in which these two variables were continuously measured as a time series. Thus, the pattern of the WD-LI space reflected the participant's visual behavioral track in a specific observation period. The “static” characteristic of the pattern was summarized in a heatmap, in which the space was divided into 40 × 40 pixels in the range of 0 to 70 cm for WD and logarithm of 0 to 10^5^ lux for LI (Fig. 2B). Each pixel in the heatmap actually represented one specific circumstance where visual behavior occurred, and the concentration of the pixel, indicated by the color, represented the percentage of time (PoT) spent in the corresponding circumstance during the observation period. For one subject, in each pixel of WD-LI space, the PoT is the ratio of the time falling into the pixel to the total measured time of the subject. If no time falls into the pixel, the PoT in the pixel is 0. Thus, the sum of all grids in the heatmap equals to 1.

#### Step 3: Dealing with Sparsity in WD-LI Measurements

The WD-LI measurements in the population can be put in a behavior-function context, in which the neighboring pixels of the WD-LI space form behavior information while SER acts as function information. The core target of the analysis is to quantify the WD-LI behavior in relationship with the function measure (e.g., SER). For measurement that is continuous in both behavior and function space, simple regression analysis between the two would provide a good estimate of such dynamics. However, the WD-LI behavior measurements for each individual are sparse in WD-LI space. For example, Figure 2 shows the WD-LI behaviors of all subjects in the space. The empty pixels indicate that there is no behavior in the pixels (i.e., there is no PoT). To address the sparsity in the space, for each pixel, we need to “borrow” the information from the neighboring behavior. We use the two-dimensional Gaussian kernel function^16^ (1) to delimit the neighbors and assign the weight for every neighbor of the pixel located at (*i*, *j*).

(1) $$N\left\{\left(i\#,j\#\right)|\left(i,j\right),\sigma \right\}=exp\left\{\frac{-{\left|\left|\left(i\#,j\#\right),\left(i,j\right)\right|\right|}^{2}}{2\sigma^{2}}\right\},$$

where *i* = {0, 1, …, *N* − 1} represents the index on the horizontal axis and *j* = {0, 1, …, *N* − 1} represents the index on the vertical axis, *N* = 40. The hyperparameter σ controls the width of the Gaussian kernel. The pixels in the circle with the radius 2σ are considered the neighboring pixels of the pixel. (i*, j*) represents the index of neighboring pixels of the pixel (*i*, *j*) in the circle, that is, (i*, j*) = {(0, 0),  (0, 1),  (1, 0),  (1, 1),  …}, where (i*)^2^ + (j*)^2^ ≤ (2σ)^2^. (i#,j#) represents one element of (i*, j*). || .|| is the Euclidean distance. The weighted values of all the neighboring pixels of the pixel (*i*, *j*) are represented by (2):

(2) $$\begin{array}{ccc} w_{ij} & = & \{N\left\{\left(i\#,j\#\right)|\left(i,j\right),\sigma \right\}\;|\;\left(i\#,j\#\right)\;\in \;\left(i*,j*\right),\\ & & \;{\left(i*\right)}^{2}+{\left(j*\right)}^{2}\leq {\left(2\sigma \right)}^{2}\}. \end{array}$$

## Data Analysis

### Relationship between the WD-LI Space and Refractive Error

Individual refractive error was expressed by SER. To take advantage of the different dependencies between the pixel and the neighboring pixels in the spatial space, we performed weighted linear regression on each pixel by (3):

(3) $$\mathrm{SER}\;=\beta_{ij}w_{ij}X+b.$$

***SER*** and ***X*** represent the SER and PoT of all subjects, respectively. β_*ij*_ describes the relationship between ***X*** and ***SER***.

### Proposing a VBI

Further, we proposed a novel parameter VBI to reflect the overall effect of visual behavior of a subject *m* over a period on the SER. As mentioned above, the percentage of time that the subject *m* spent in one pixel of the heatmap to the entire visual behavior time was PoT_*m*_, and the influence of each pixel (*i*, *j*) on the SER was **B***^ij^* (**B***^ij^* = β_*ij*_, if P*_ij_* < 0.05; **B***^ij^* = 0, if P*_ij_* ≥ 0.05). Therefore, the PoT_*m*_ of each pixel passed by the subject *m* and the corresponding B*^ij^* were multiplied and then accumulated to generate the novel parameter VBI, which can reflect the overall effect theoretically. Its calculation formula is as (4):

(4) $$VBI_{m}=\sum_{j=0}^{N-1}\sum_{i=0}^{N-1}PoT_{m}^{ij}\;*\;B^{ij}.$$

Additionally, we used linear regression to verify the relationship between VBI and ***SER***.

## Results

The study included a total of 179 fourth-grade students with a mean ± standard deviation (SD) age of 9.17 ± 0.52 years, and 92 (51.40%) were boys. All participants underwent a cycloplegic autorefraction, the mean ± SD SER was 0.22 ± 1.18 D, and 18.44% (33/179) were myopic.

**Figure 3.**
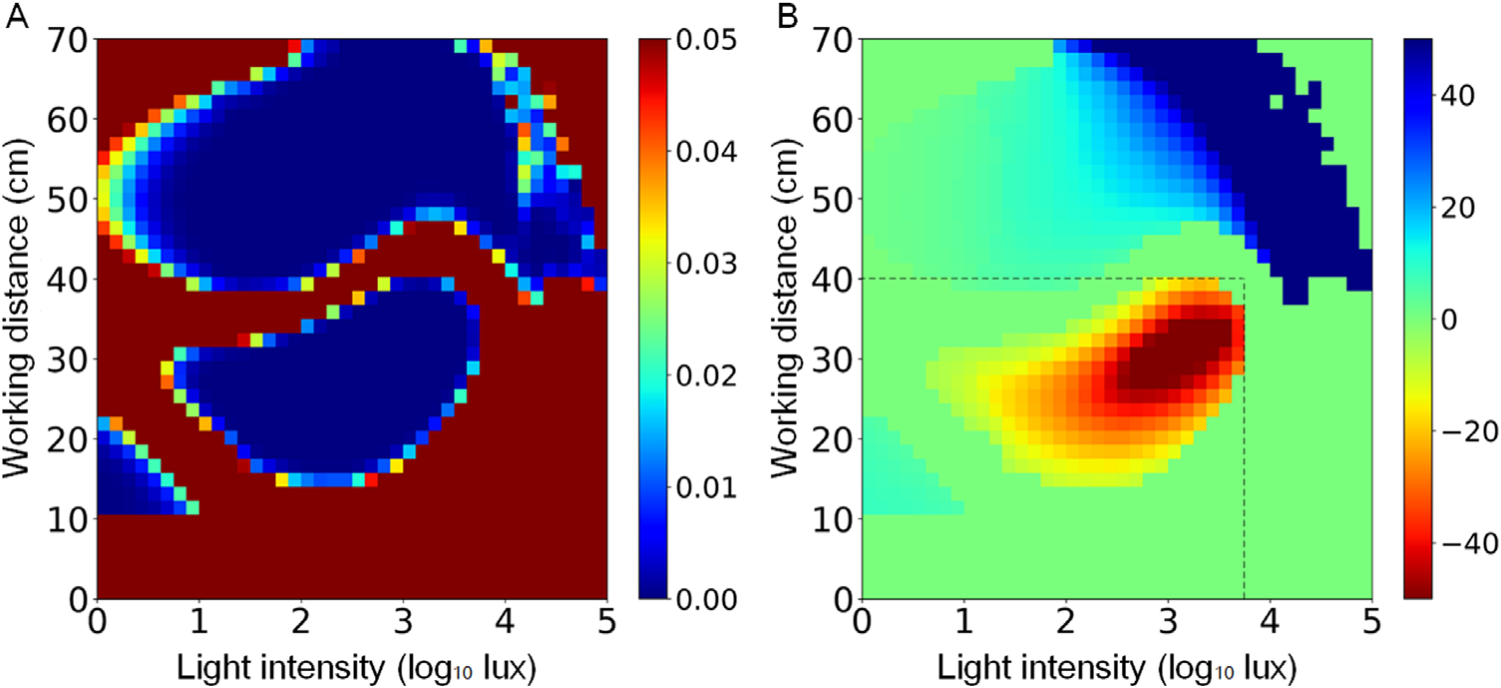
Illustrations of the P value map (A) and the strength (β value) map (B). In the P value map, the *warmer* (*red*) the color, the larger the P value, and the *cooler* (*blue*) the color, the smaller the P value. In the strength map, *warm color* indicates a negative correlation between the visual behavior and refractive error (e.g., the longer time to stay in this circumstance, a trend to a higher degree of myopic SER). *Cool color* indicates a positive correlation between the visual behavior and refractive error (e.g., the longer time to stay in this circumstance, a trend to a higher degree of hyperopic SER). *Green* in the strength map indicates no significant correlation between the visual behavior and refractive error. The horizontal and vertical dashed lines represent the upmost and rightmost border of the risky zone, respectively.

Figure 3 illustrates the probability (P) and the strength (β) of the correlation between each pixel of the WD-LI space and refractive error, obtained from the weighted linear regression. It is observed that in general, shorter WD and lower LI manifested a detrimental effect on refractive error toward myopia direction, except for some circumstances where there is no statistically significant correlation, probably due to insufficient visual behavior occurring there. However, the strength of the impact of these two environmental factors on refractive error varied with the relative level between them. If we split up the WD and LI, the upmost limit of the red area with statistical significance (i.e., detrimental effect related to myopia) was about 40 cm for WD, and the rightmost limit of the red area with statistical significance was about anti-log_10_ 3.8 (i.e., 6300 lux). This indicates that for a working distance larger than 40 cm, near work does not exert a detrimental effect on the refractive error toward myopia direction regardless of the eye-level light intensity. Similarly, for an eye-level light intensity larger than 6300 lux, near work does not exert a detrimental effect on the refractive error toward myopia direction regardless of the working distance. Otherwise, for a circumstance in which working distance is less than 40 cm or eye-level light intensity is less than 6300 lux, the final impact of one environmental factor depends on the level of the other environmental factor. Under the light intensity between the scotopic and mesopic level (i.e., less than 10 lux), working distance shorter than 20 cm manifested a modest protective effect against myopia.

**Figure 4.**
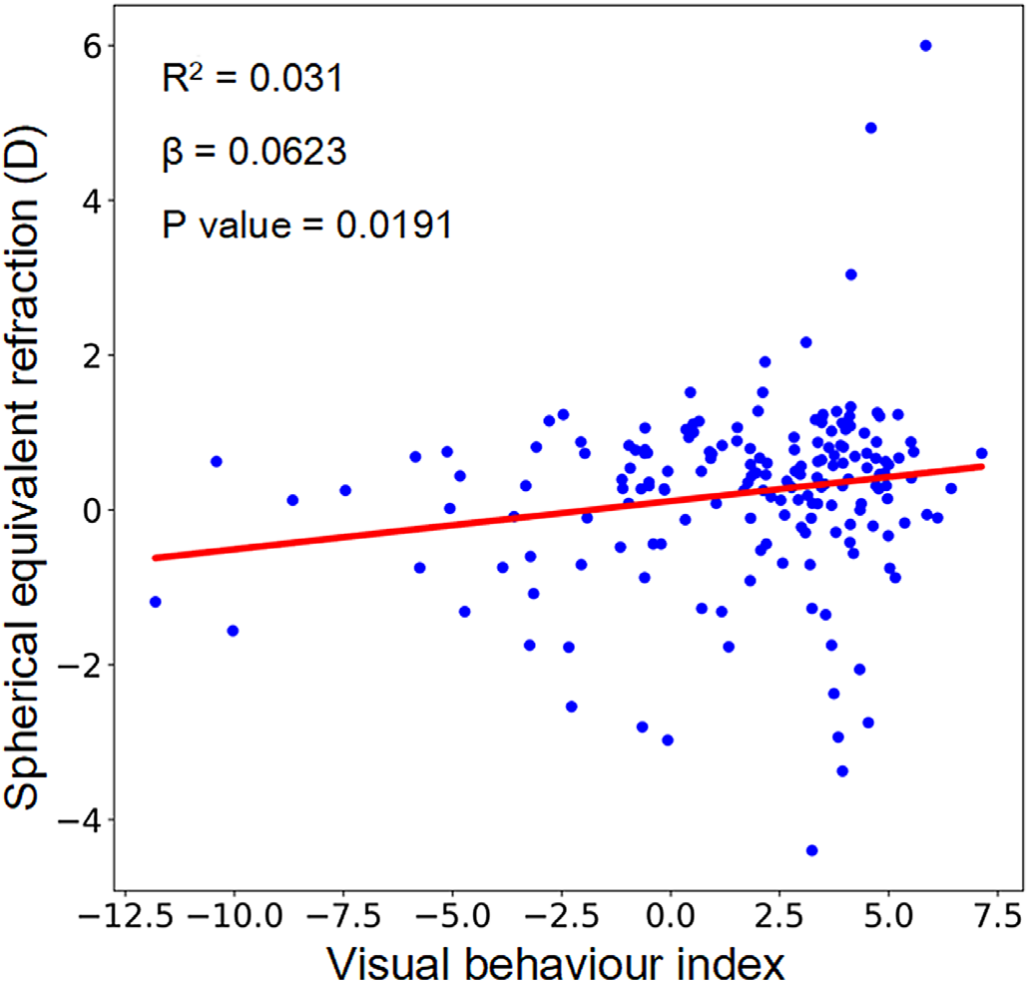
Illustration of the results of the ordinary linear regression between VBI and SER for all subjects. The VBI and SER were significantly positively related.

The ordinary linear regression was used to explore the relationship between the VBI and SER, and the result is illustrated in Figure 4. The mean ± SD of the VBI was 1.50 ± 3.36, and the range was (−11.53 to 6.58). The VBI and SER were significantly positively related (β = 0.0623, *R*^2^ = 0.031, *P* < 0.05). When the VBI increased, the SER moved toward hyperopia, and when the VBI decreased, the SER moved toward myopia.

## Discussion

In this study, we used the theories and algorithms of spatial data mining to explore the association between two well-recognized environmental risk factors, WD and LI, and refractive error. Systematic methods were adopted, including spatial data classification, spatial neighboring relations, and grid-based clustering algorithms. Furthermore, we visualized these results to make them more intuitive and understandable. Compared with the traditional approaches, our approaches extended from the two single-dimensional environment factors WD and LI to the two-dimensional WD-LI space that covers almost all possible visual behavior features in a time series. In this two-dimensional space, for each pixel, we explored the protective and detrimental effect of visual behavior on SER in a fine-grained manner. To our best knowledge, this is the first time a study has quantified the environmental risk factors in such a two-dimensional space, so we were able to investigate the comprehensive influence of WD and LI on SER in a weighted manner. We have set different weights to different WD and LI, which makes it more accurate to explore the impact of both on SER. Finally, a single parameter that summarized the overall effect of the aforementioned environmental data of a subject, VBI, was proposed to make this approach feasible by clinicians in practice.

Using our novel approach for evaluating the effect of WD and LI on refractive error simultaneously, we found that the effect of these two environmental factors on SER varied with the relative level between them. An “absolute” threshold of working distance could be defined, if we separated the two factors. In this group of population, we found working distance that was greater than 40 cm seems to be safe from myopia. Considering working distance alone, previous studies have proposed varied thresholds. For instance, Ip et al.^17^ reported that less than 30 cm of working distance was detrimental for myopia. In another study, Li et al.^18^ showed that less than 20 cm had a dangerous effect on myopia. Thresholds of these studies were mainly quantified through questionnaires, and the threshold in the quantification was usually set by the researchers manually. The questionnaires typically asked whether the subject frequently, occasionally, or never performed near work within a specific distance to determine the distance threshold for myopia. In addition, the respondents of questionnaires usually had a difficult time estimating their habitual working distance as well as the time duration at this distance accurately. Thus, a specific threshold of WD through questionnaire approaches is unlikely to be defined. In contrast, the current study adopted Clouclip to record the WD in a real-time manner and treated the distance data in a continuous fashion. The resultant threshold is theoretically more convincing.

Thanks to the available wearable devices, several studies have attempted to investigate the role of light intensity on refractive error in an objective manner.^19^^,^^20^ Read et al.^19^ adopted Actiwatch to measure LI and found that more than 3000 lux was a threshold for LI, which has a protective effect against myopia. Through another wearable device, HOBO, Wu et al.^20^ found that LI of approximately >1000 lux can reduce the risk of myopia development by 35%. In our study, the threshold of LI was 6300 lux if the impact of WD was excluded, which suggests that light intensity greater than this level would provide a protective effect regardless of the reading distance. It should be pointed out that when spending time outdoors with a viewing distance of 70 cm or more, the threshold needed may be much less. Although the threshold of effective LI differed between these studies, it is very difficult to compare the values directly due to the different study designs and devices used in these studies. HOBO is generally worn on the shirt (collar or chest) with the light sensor facing outward. Actiwatch is a wrist-worn device with a direction of measurement that depends on the wrist positioning. However, Clouclip is a spectacle-mounted device that measures LI along the line of sight. The different locations and directions where the sensors face would have led to differing levels of the light intensity. In addition, as mentioned earlier, the previous studies usually treated one variable alone, while the current study analyzed both LI and WD simultaneously.

Interestingly, the left bottom corner of Figure 3 indicates that WD <20 cm was moderately protective for myopia. This finding seems to contradict previous studies showing that this level of near-working distance was a risk factor for myopia. It should be pointed out that the association determined in Figure 3 should be interpreted in the context of the two-dimensional analysis approach. The protective effect observed in the present study was not the effect of the near-working distance alone but rather the combined effect of both the near-working distance and light intensity. It is believed that the protective effect shown is more likely attributable to the dim-light intensity in the scenario, because a recent study revealed that dim-light exposure, similar to bright-light exposure, was also important for myopia inhibition.^21^

The overall effect of the aforementioned environmental data of a subject could be summarized as a single parameter, VBI. The linear regression analysis further confirmed the association between VBI and individual refractive error with a *R*^2^ of 0.031. To our knowledge, this is the first time a study has shown the attributable impact of these two environmental factors on individual refractive error. Therefore, it is difficult to judge whether the impact is high or low, but a recent meta-analysis investigating the heritability of refractive error explained by common genetic variants reported the impact of genetic background was at a similar level (i.e., heritability = 0.053) in the Asian samples.^22^ It should be pointed out that the refractive error measured actually represents, if any, the consequence of prior exposure to environment risk factors, while the data obtained by Clouclip are the data that would affect refractive error in the future. Thus, prospective studies are needed to evaluate the effect of VBI on the refractive error.

Obtaining thresholds of these two major environmental factors may help provide relevant references for environmental modifications with regard to myopia control. Nevertheless, the specific level of the thresholds should not be applied to practice directly because of the limitations of the study. First, although the study covered the activities performed on both weekdays and weekend days, and the students’ schedules of activities tended to be relatively regular, the Clouclip device was worn for only 1 week, which might not fully represent the visual behavior of the subjects for a longer period. Second, the relatively small sample size in the current study may not be sufficient to accurately assess the quantitative relationship between environmental factors and refractive error. In addition, given the nature of the cross-sectional design of the study, the thresholds of these two major environmental factors as well as the *R*^2^ of VBI warrant further refinement through prospective studies. In light of this, this group of participants is currently undergoing follow-up procedures through a longitudinal study to help us better understand their associations.

In conclusion, we confirmed the association between working distance, light intensity, and refractive error through a novel approach based on the combination of a wearable device and big data science. Individuals’ risk of myopia due to exposure to these two major environmental risk factors could be further summarized into a single parameter of VBI. Our novel approach might provide a useful tool to investigate the role of environmental risk factors in myopia research, as well as a practical tool to predict myopia development and progression in practice.
